# P-1407. The Growing Trend of Clinical Tick-Borne Diseases in South Carolina

**DOI:** 10.1093/ofid/ofae631.1582

**Published:** 2025-01-29

**Authors:** Kayla E Bramlett, Emily Owens Pickle, Hanna Waltz, Eleanor Saunders, Julia Vorobiov, Scott Commins, Matthew Haldeman, Melissa Nolan

**Affiliations:** University of South Carolina, Simpsonville, South Carolina; University of South Carolina, Simpsonville, South Carolina; University of South Carolina, Simpsonville, South Carolina; University of North Carolina at Chapel Hill, Chapel Hill, North Carolina; University of North Carolina at Chapel Hill, Chapel Hill, North Carolina; University of North Carolina at Chapel Hill, Chapel Hill, North Carolina; University of South Carolina, Simpsonville, South Carolina; University of South Carolina, Simpsonville, South Carolina

## Abstract

**Background:**

The trend of human tick-borne disease (TBD) incidence has increased exponentially over the past two decades. Limited healthcare access, substandard housing, socioeconomic fragility, and absence of government-backed tick control initiatives significantly contribute to the high disease burdens nationwide. Given the wide prevalence of socioeconomic and health inequalities in the USA, the risk of TBDs often goes undetected in impoverished and minority communities. Alpha-gal syndrome (AGS) is one TBD that is often overrepresented in white, middle-income individuals, and little information on the incidence in populations of Color exists. Therefore, the current study aims to estimate the seroprevalence and exposure-associated risk factors of AGS among vulnerable populations in South Carolina.

**Methods:**

First, a five-year retrospective clinical chart abstraction of AGS patients within a large healthcare system covering half of South Carolina was completed to characterize the clinical burden and natural history of AGS. Next, banked serum samples and associated surveys from a cross-sectional minority-represented COVID-19 study were analyzed for AGS seropositivity.

**Results:**

Fifty patients with positive AGS IgE labs were identified via retrospective chart review. Of these, the majority were male (70%), covered by Medicare (84%), and had a history of tick bites or spent extended periods outdoors (46%). The cross-sectional AGS seroprevalence study detected 3.5% seropositivity among 753 serum samples. Risk factors associated with a positive titer included being male (58%), and regularly spending time outdoors (58%). Notably, the seroprevalence cohort consisted of primarily Black residents and individuals who live below the poverty line.

**Conclusion:**

These findings highlight the growing issue of TBDs, namely AGS, among minority populations in South Carolina, urging healthcare providers to be more informed.

**Disclosures:**

**All Authors**: No reported disclosures

